# Observations of a novel predatory gull behavior on an invasive ascidian: A new consequence of coastal urban sprawl?

**DOI:** 10.1002/ecs2.2636

**Published:** 2019-03-07

**Authors:** Luke E. Holman, Marc Rius, Tim M. Blackburn

**Affiliations:** ^1^ School of Ocean and Earth Science National Oceanography Centre Southampton University of Southampton Southampton UK; ^2^ Centre for Ecological Genomics and Wildlife Conservation University of Johannesburg Johannesburg South Africa; ^3^ Centre for Biodiversity and Environment Research Department of Genetics, Evolution and Environment University College London London UK; ^4^ Institute of Zoology Zoological Society of London London UK

**Keywords:** avian cognition, biotic resistance, foraging innovation, invasive, non‐native, urban sprawl

## Abstract

Coastal urbanization has a dramatic effect on both terrestrial and marine ecosystems, altering resources such as food or space. Many species have shifted their ranges in response to anthropogenic pressures, resulting in novel species interactions. Here, we report an observation of a novel foraging behavior of the European Herring Gull (*Larus argentatus*): the capture and consumption of the widespread sea squirt *Ciona intestinalis* from under floating pontoons in a recreational marina in Ireland. Multiple gulls were observed performing a complex, multi‐step manipulation of several *C. intestinalis* individuals to remove their cellulose‐based tunic, which remained unconsumed. Further avenues of investigation are discussed, and hypotheses concerning possible ecosystem effects of novel ecological interactions occurring in proliferating artificial environments are presented.

## Introduction and Observations

Seabirds in the family *Laridae* (gulls) are without exception generalist feeders and well known for their adaptability in exploiting novel sources of food (Burger and Gochfeld 1996). This opportunism has become increasingly relevant in recent centuries, as humans have dramatically altered coastal habitats around the world where most gull species commonly reside (Firth et al. 2016). Many of the gull species living along coastlines have learned to exploit anthropogenic food sources, notably rubbish dumps and bins (Horton et al. 1983), with some bold individuals even learning to enter shops or swoop at humans to take food (Deering 2017). Here, we report a novel feeding association that seems to have developed as a consequence of these changes—predation by European Herring Gull (*Larus argentatus*) on the widespread sea squirt *Ciona intestinalis*.

**Figure 1:**
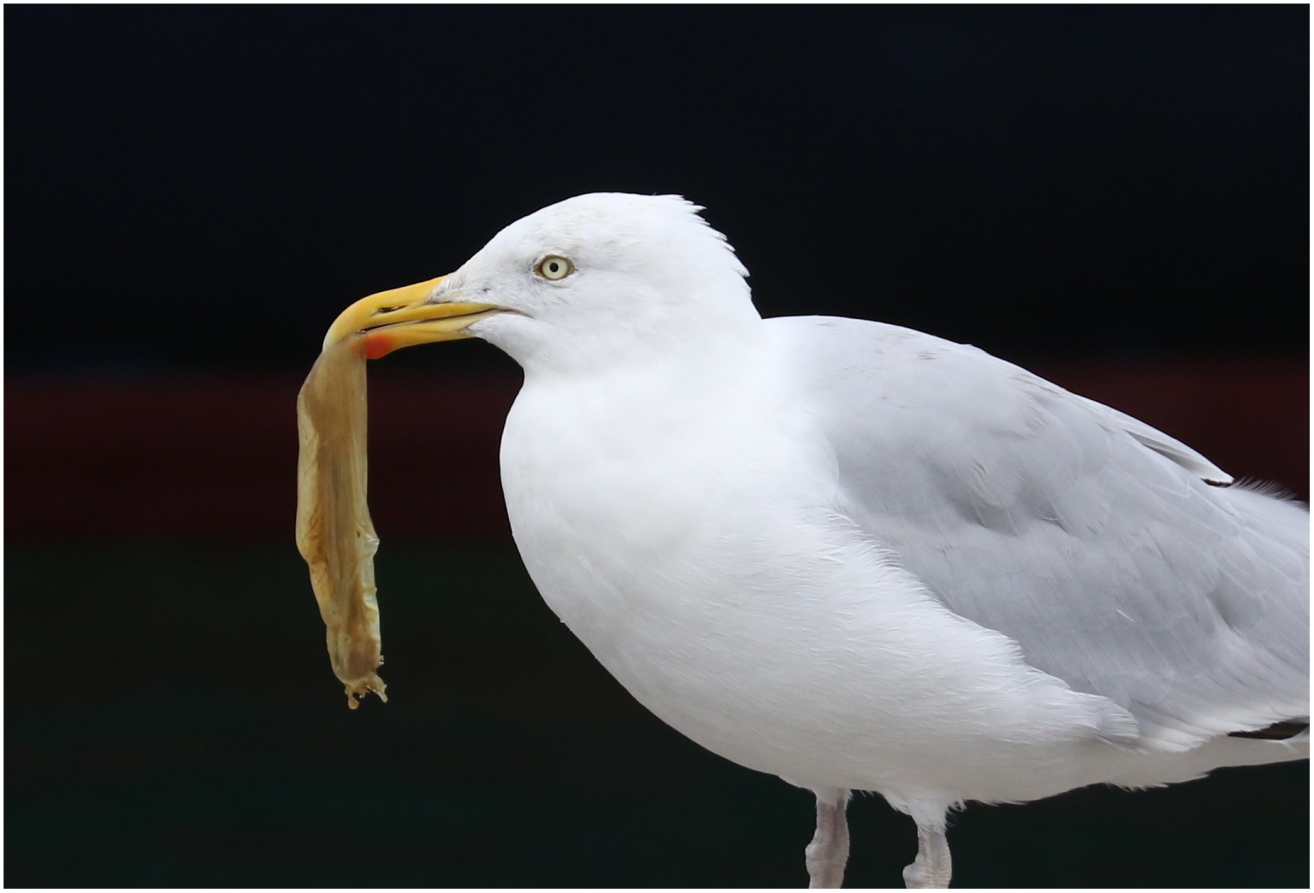
European Herring Gull (*Larus argentatus*) clasping a *Ciona intestinalis* individual in Dún Laoghaire Marina, Ireland. Photo: T. M. Blackburn.

**Figure 2:**
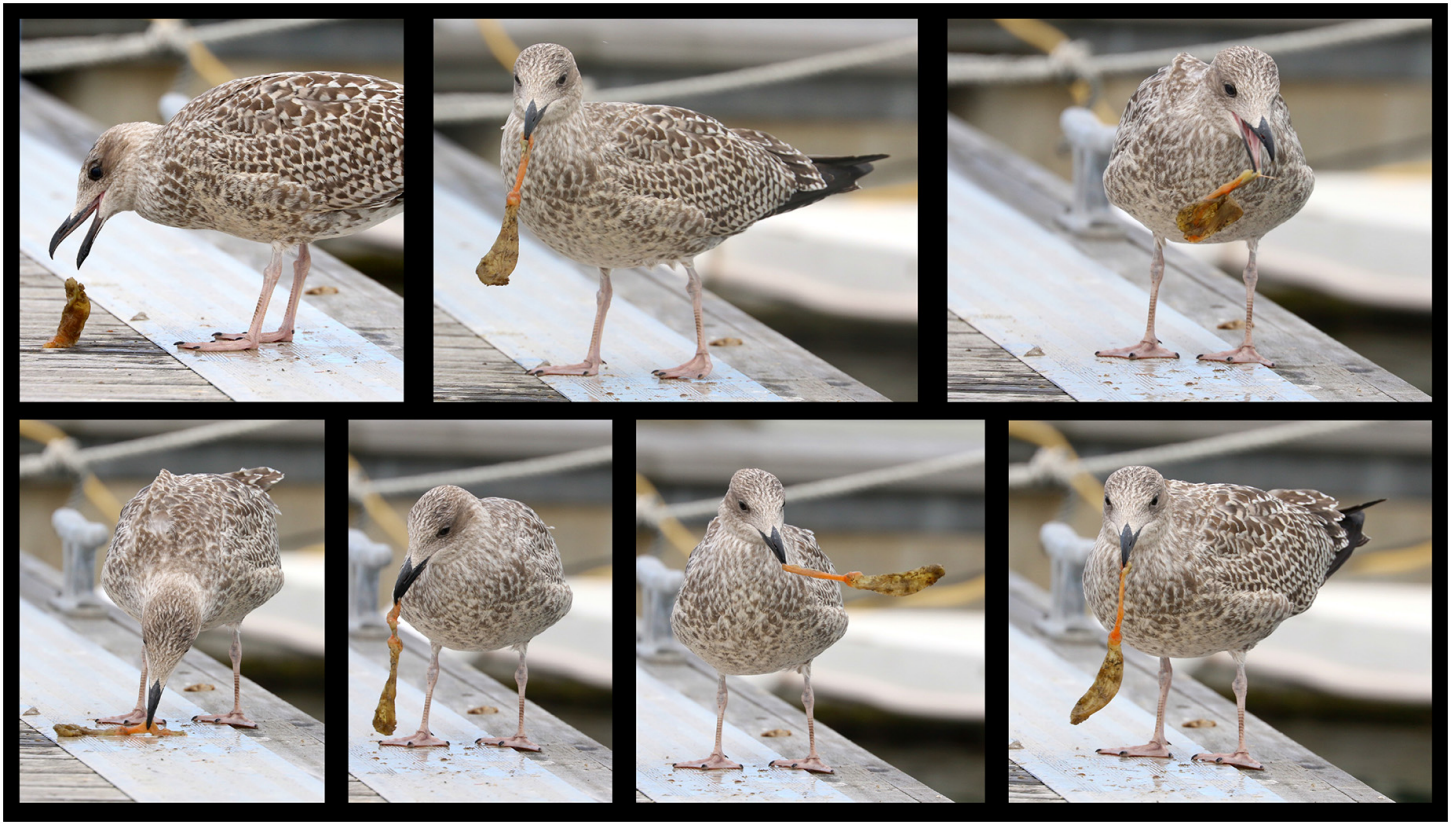
Sequence detailing the method employed by Herring Gulls in Dún Laoghaire Marina to strip *Ciona intestinalis* of the outer tunic. Photo: T. M. Blackburn.

On the 5th of September 2018, we visited the marina in Dún Laoghaire harbor, Ireland, conducting surveys for marine non‐indigenous species. As we walked along the floating dock between the rattling masts of the numerous yachts moored there, we witnessed a Herring Gull diving down below the pontoons and emerging grasping a translucent soft object (Fig. 1). Upon closer observation, the gull appeared to have retrieved a sea squirt of the species *C. intestinalis* from its position hanging under the submerged floating pontoon. Sea squirts or ascidians (Class Ascidiacea, Phylum Chordata) are sessile filter‐feeding organisms that have a soft inner body and an outer layer known as a tunic, which serves as protection from predators and fouling organisms. The gull subsequently settled on a pontoon where it proceeded to manipulate the sea squirt, removing the tunic using the decking as a working station. As shown in Fig. 2 (illustrated with a second gull), the method employed by the gull was to grasp the sea squirt in its beak, holding the posterior end, and then shake the animal to loosen its soft inner body. As the inner body loosened, the gull dropped the sea squirt and switched its hold to the inner body and continued to shake until the body came entirely free. It took no more than 20 seconds for the gull to have completely separated the ascidian body from its tunic, after which the gull then proceeded to swallow the main body. As we continued through the marina, we observed several (between 5 and 15) *L. argentatus* individuals fishing for ascidians, either by diving or by simply sitting on the water surface and ducking for sea squirts that had opted to settle on shallow sections of the pontoons. During our observations, we noticed abundant evidence of this feeding method in the form of a large number of discarded tunics, many dried out. We had never observed gulls feeding on or manipulating sea squirts that inhabit marinas in our years of fieldwork and birdwatching in those habitats across the globe.

Separating ascidians from their tunic is necessary for morphological analysis of characteristics that are frequently diagnostic of the ~3000 known extant ascidian species (Appeltans et al. 2012), but it is a task that requires practice to master. It was therefore a surprise to find that a gull had acquired a competency usually reserved for ascidian taxonomists, especially on a species like *C. intestinalis* where the tunic is tightly connected to the main body. Several other gull species were observed in the marina, including Mediterranean (*Larus melanocephala*), Black‐headed (*Chroicocephalus ridibundus*), Lesser Black‐backed (*Larus fuscus*), Great Black‐backed (*Larus marinus*), and Black‐legged Kittiwake (*Rissa tridactyla*), but only *L. argentatus* were observed feeding on *C. intestinalis*. Turnstones (*Arenaria interpres*) were also present on the pontoons, but despite their famously varied diet (Gill 1986) were only observed feeding on *L. argentatus* droppings (cf. King 1982), and not on discarded *C. intestinalis* tunics or individuals.

It is not clear why the ascidian tunic is unpalatable to the gulls, but studies have shown that in some ascidian species the tunic is highly acidic (Parry 1984), while in others it may contain high levels of vanadium (Stoecker 1980). Palatability trials have not indicated that the tunic of *C. intestinalis* contains any detectable chemical defense (Teo and Ryland 1994). The most probable explanation is that the gulls are simply unable to digest the cellulose‐containing tunic and choose to discard it. Indeed *L. argentatus* has a markedly short cecum, the length of which is positively correlated with herbivory and the digestion of cellulose (DeGolier et al. 1999). Regardless, the dexterity and handling ability exhibited by the gulls that separated the tunic from the body, both on land and while surface swimming, is unprecedented.

**Figure 3:**
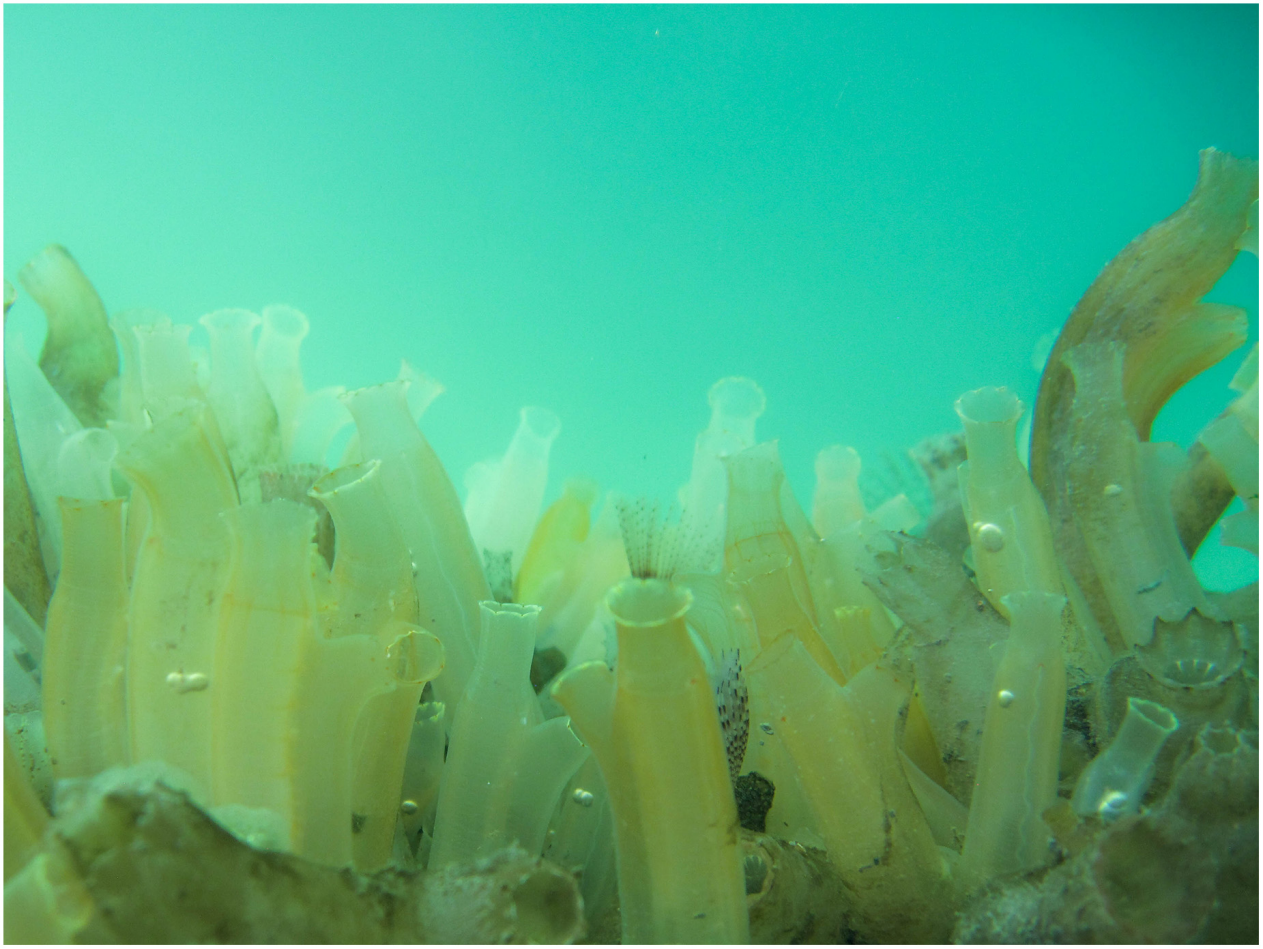
An epibenthic community in Northney Marina, UK, demonstrating ecological dominance of the ascidian *Ciona intestinalis*. Photo: L. E. Holman.

*Ciona intestinalis* is a highly successful fouling organism and, like the Herring Gull, has benefited from coastal urbanization (e.g., marinas and harbors) and artificial transport of species (e.g., transoceanic shipping and recreational boating). Recent taxonomic studies have revised the *C. intestinalis* species complex confirming *Ciona robusta* (previously *C. intestinalis* type A) and *C. intestinalis* (previously *Ciona intestinalis* type B; Brunetti et al. 2015) as seperate species. *C. intestinalis* is the subject of these observations and has a range across coastlines of Europe and North America (Bouchemousse et al. 2016). Floating pontoons are often dominated by *C. intestinalis* (Fig. 3), which is able to grow extremely rapidly and competitively exclude many other sessile invertebrates (Collin and Johnson 2014). Like most organisms living in the ocean, ascidians face numerous marine predators throughout their ontogeny (Dumont et al. 2011), and previous studies have identified ascidians in the diets of other gull species. For example, analysis of the stomach contents of twelve Glaucous Gulls (*L. hyperboreus*) reported by Burton and Thurston (1959) revealed a trace of an ascidian test, possibly from a *Ciona* species, in a gull stomach. Divoky (1976) identified a pyurid ascidian in the stomach contents of one of thirteen Ivory Gulls (*Pagophila eburnea*) collected in the Arctic Chukchi Sea. Despite these observations, our report represents the first record of Herring Gulls acting as predators on *C. intestinalis*, suggesting that ascidians form a significant source of food for the Herring Gulls of Dún Laoghaire harbor. Our observations reinforce the general view that *L. argentatus* is a highly versatile predator and corroborates existing work identifying cognitive handling of objects in the foraging of gull species (Henry and Aznar 2006).

## Remaining Questions

The observations recorded here prompt many avenues for further investigation. Key will be determining the frequency and seasonality of gull behavior in Dún Laoghaire and understanding to what extent it is unique to the area. It will be important to learn how or whether this behavior has spread to other areas, and to unravel the mechanism through which it may have propagated through other populations. Additionally, understanding what proportion of the gull's diet is derived from feeding on the sea squirts, and whether this change exerts pressure on alternative sources of natural prey, will help contextualize this behavior into the wider ecology of the system.

In isolation, a novel animal behavior like the one reported here is fascinating, but in this case, the possible implications at the local, regional, and global levels suggest some intriguing hypotheses. Could the artificial spread of *C. intestinalis* facilitate both population growth and range expansion in *L. argentatus,* as seen in the African Black Oystercatcher (*Haematopus moquini*) in response to the invasion of the Mediterranean Mussel (*Mytilus galloprovincialis*) along South African coastline (Branch and Nina Steffani 2004)? Herring Gull is on the Red List of the Birds of Conservation Concern 4 (Eaton et al. 2015) because of severe, long‐term population declines in the UK, and as such might benefit from such an innovation. What implications does relying on *C. intestinalis* have for the gull population over time? As *C. intestinalis* population size can oscillate across years (Dybern 1965), how might a decrease in food availability affect the ecology of the local terrestrial environments? Finally, disturbance is known to facilitate the establishment of non‐indigenous species along urbanized coastlines (Clark and Johnston 2009), and so how will the feeding of the gulls on *C. intestinalis* affect the ability for novel non‐indigenous species to colonize these environments? Future research should tackle these and other pressing questions in a world where the construction of marinas and harbors is on the rise.
